# Impact of Arginine to Cysteine Mutations in Collagen II on Protein Secretion and Cell Survival

**DOI:** 10.3390/ijms19020541

**Published:** 2018-02-11

**Authors:** Salin A. Chakkalakal, Juliane Heilig, Ulrich Baumann, Mats Paulsson, Frank Zaucke

**Affiliations:** 1 Center for Research in FOP and Related Disorders, Perelman School of Medicine, University of Pennsylvania, Philadelphia, PA 19104, USA; salin.chakkalakal@gmail.com; 2 Center for Biochemistry, Medical Faculty, University of Cologne, 50931 Cologne, Germany; juliane.heilig@uni-koeln.de (J.H.); mats.paulsson@uni-koeln.de (M.P.); 3 Cologne Center for Musculoskeletal Biomechanics (CCMB), 50931 Cologne, Germany; 4 Institute of Biochemistry, University of Cologne, 50931 Cologne, Germany; ubaumann@uni-koeln.de; 5 Cluster of Excellence Cellular Stress Responses in Aging-Associated Diseases (CECAD), University of Cologne, 50931 Cologne, Germany; 6 Center for Molecular Medicine Cologne (CMMC), University of Cologne, 50931 Cologne, Germany; 7 Dr. Rolf M. Schwiete Research Unit for Osteoarthritis, Orthopedic University Hospital Friedrichsheim, 60528 Frankfurt/Main, Germany

**Keywords:** collagen II, chondrodysplasia, mutation, unfolded protein response, triple helix

## Abstract

Inherited point mutations in collagen II in humans affecting mainly cartilage are broadly classified as chondrodysplasias. Most mutations occur in the glycine (Gly) of the Gly-X-Y repeats leading to destabilization of the triple helix. Arginine to cysteine substitutions that occur at either the X or Y position within the Gly-X-Y cause different phenotypes like Stickler syndrome and congenital spondyloepiphyseal dysplasia (SEDC). We investigated the consequences of arginine to cysteine substitutions (X or Y position within the Gly-X-Y) towards the N and C terminus of the triple helix. Protein expression and its secretion trafficking were analyzed. Substitutions R75C, R134C and R704C did not alter the thermal stability with respect to wild type; R740C and R789C proteins displayed significantly reduced melting temperatures (T_m_) affecting thermal stability. Additionally, R740C and R789C were susceptible to proteases; in cell culture, R789C protein was further cleaved by matrix metalloproteinases (MMPs) resulting in expression of only a truncated fragment affecting its secretion and intracellular retention. Retention of misfolded R740C and R789C proteins triggered an ER stress response leading to apoptosis of the expressing cells. Arginine to cysteine mutations towards the C-terminus of the triple helix had a deleterious effect, whereas mutations towards the N-terminus of the triple helix (R75C and R134C) and R704C had less impact.

## 1. Introduction

Collagens are the major proteins in the connective tissues, and collagen II is the major collagen type in cartilage. However, it is also expressed in the vitreous of the eye and is detected during early embryogenesis [1]. It is also expressed in nonchondrogenic tissues including notochord and several parts of the developing eye [2]. Structurally, collagen II consists of three α chains, which are wound around each other into a right-handed triple helix, which consists of repeats of three amino acids (Gly-X-Y repeats). In various heritable connective tissue disorders, glycine substitutions in the collagen chains cause structural changes that result in reduced thermal stability [3,4,5]. 

Cells synthesize collagen II α chains as longer precursors called procollagens. Simultaneously, the growing polypeptide chains are co-translationally transported into the rough endoplasmic reticulum (ER) where they undergo a series of post-translational modifications [6]. In addition to post-translational modifications, the ER performs a stringent quality control for unfolded molecules, and these are either retained or degraded. The correct folding and assembly of proteins within the endoplasmic reticulum (ER) are prerequisites for subsequent transport from this organelle to the Golgi apparatus [7]. Only recently, the pathway of collagen secretion from the Golgi complex to the plasma membrane in large cargo vesicles has been elucidated [8]. The recognition and retention of unassembled or misfolded proteins requires an interaction with molecular chaperones within the ER [9]. One classic example of this process occurs during the biosynthesis of procollagen. Incompletely folded intermediates or misfolded products are recognized by chaperones that prevent their secretion and eventually lead to intracellular accumulation [10]. Procollagens are secreted into the extracellular matrix (ECM) through the Golgi apparatus. The collagen II structure is susceptible to mutations as it involves this complex process of collagen synthesis, folding and assembly, leading to disease states that are collectively termed chondrodysplasias and comprise a wide range of well-characterized clinical phenotypes [11,12,13]. Most chondrodysplasias are due to point mutations in the gene encoding collagen II. Glycin replacement within the Gly-X-Y repeats accounts for 34% of all mutations [14]. These changes result in abnormal conformation and destabilization of the triple helix [15]. Only a few non-glycine missense mutations have been reported, and among these, the arginine to cysteine substitutions predominate [16]. 

Point mutations leading to a change from arginine to cysteine are interesting since they produce a broader spectrum of unusual phenotypes with either normal or short stature, but never lethal conditions, i.e., congenital spondyloepiphyseal dysplasia (SEDC), Stickler syndrome, Czech dysplasia metatarsal type and osteoarthritis-associated SED. Different amino acid substitutions in the X position of Gly-X-Y repeats have been shown to cause variable phenotypes in Stickler syndrome [17], while it was speculated that substitutions in the Y position might lead to SEDC.

In the present study, we analyzed the impact of arginine to cysteine mutations at the protein level and their effect on intracellular trafficking and secretion. A panel of mutations towards the N- (R75C, R134C) and C- (R704C, R740C and R789C) terminus of the triple helix was selected. These mutations cause a spectrum of different clinical phenotypes in humans including Czech dysplasia metatarsal type (R75C) [18]), Stickler syndrome (R704C) [19,20] and spondyloepiphyseal dysplasia congenita (R789C) [21]. All of these mutations are located in the triple helical region and in the X or Y position of the Gly-X-Y repeats. In addition to these mutants, two artificial mutations R134C and R740C, which are not naturally identified, but lying in the X position of a Gly-X-Y repeat in the triple helix, were also studied.

## 2. Results

### 2.1. Collagen II Mutations towards the C Terminus Affect Secretion and Increase Susceptibility to Proteases 

**Figure 1:**
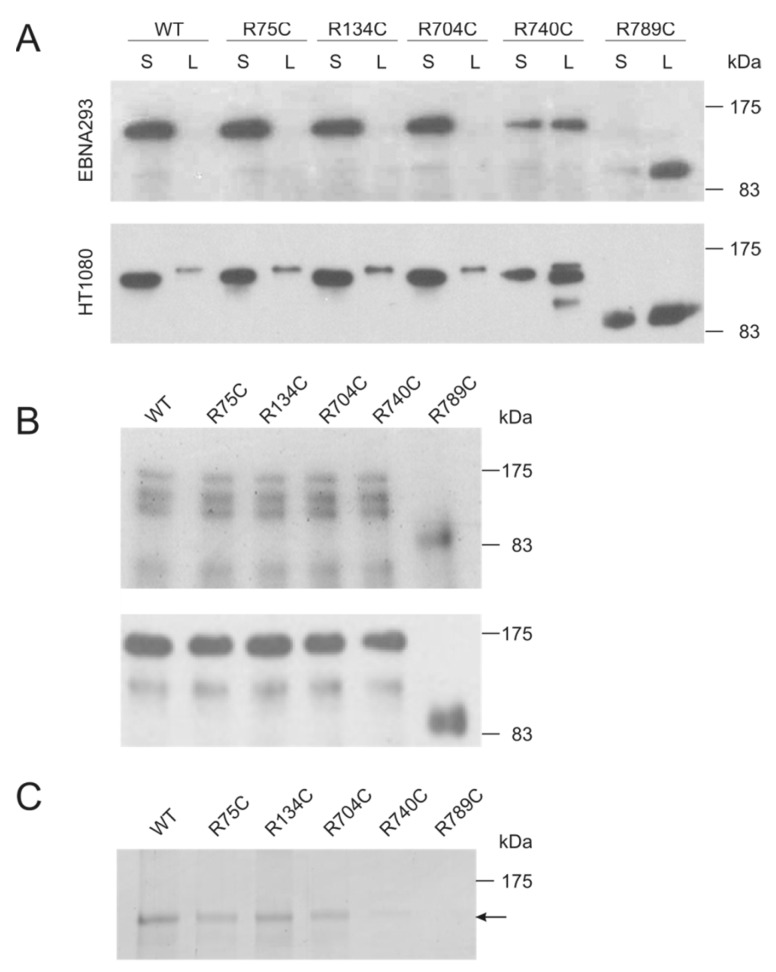
Recombinant expression of collagen II variants. (**A**) 293 Epstein–Barr nuclear antigen (EBNA) (upper panel) and HT1080 cells (lower panel) were transfected with collagen II constructs (WT, R75C, R134C, R704C, R740C and R789C) and the expression and secretion was analyzed using immunoblots. Supernatants (S) and cell lysates (L) were harvested three days post-transfection and separated by SDS PAGE (8% gel) under reducing conditions. Collagen II was detected with an antibody directed against the myc epitope. (**B**) Coomassie blue stained SDS-PAGE gel and immunoblot of purified collagen II variants using an antibody directed against collagen II (upper panel) and the myc epitope (lower panel). (**C**) Collagen II variants were treated with trypsin at 25 °C for 2 min followed by SDS-PAGE. The collagens were visualized by silver staining. WT, as well as R75C, R134C and R704C collagens were resistant to trypsin and displayed a single band of the size of the triple helical domain without the N- and C-propeptides (indicated by an arrow), whereas R740C and R789C collagens were completely degraded.

Collagen II variants were detected in supernatants and cells lysates of transiently-transfected 293 Epstein–Barr nuclear antigen (EBNA) and HT1080 cells (Figure 1A). All variants were expressed in both cell lines, but R740C and R789C were not as efficiently secreted as the other proteins and found in higher amounts in the cell lysate, indicating an intracellular retention. In addition, the R789C protein migrated much faster on SDS PAGE gels, suggesting a proteolytic processing already in the cells (Figure 1A). All secreted proteins were purified using affinity chromatography on a nickel column and detectable with specific antibodies directed against collagen II and the myc epitope, respectively (Figure 1B). Purified collagen II variants were digested with trypsin to evaluate the formation of a correctly-aligned and stable triple helix. WT, R75C, R134C and R704C collagens were trypsin resistant, while R740C and R789C collagens were completely degraded (Figure 1C). This implies that the secreted R740C and R789C proteins are not forming stable triple helical structures.

### 2.2. C-Terminal Mutations (R740C and R789C) Lead to Thermal Instability of the Triple Helix

**Figure 2:**
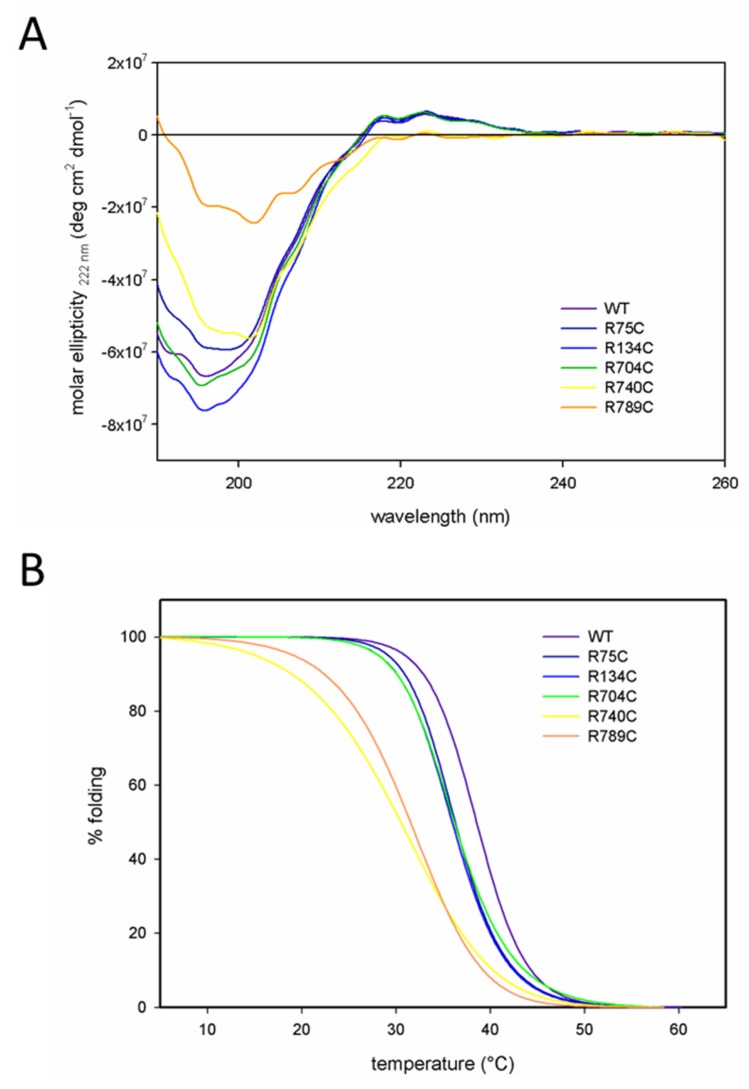
CD spectra and melting curves of purified collagen II variants. (**A**) Spectra were recorded using collagens at a concentration of 60 µg/mL after dialysis against 100 mM acetic acid. The structure of the mutant collagen II proteins R740C and R789C was altered as compared to other collagen II variants, and the shape of the spectra already indicated a decrease in triple helical structure. (**B**) Melting curves of collagen II proteins (60 µg/mL) were recorded after dialysis into 100 mM acetic acid at 222 nm with a 1 °C/min temperature gradient from 10–55 °C. To calculate the percentage of folding, a curve fit model was used assuming that initially 100% of the collagen molecules were folded. The R740C and R789C proteins displayed a decreased thermal stability when compared to all other collagen II variants.

Circular dichroism (CD) spectroscopy of WT, R75C, R134C and R704C proteins displayed typical collagen CD spectra with a positive ellipticity at 222 nm, indicating a correctly folded triple helical structure (Figure 2A). In contrast, the collagen II mutants R740C and R789C (yellow and orange line) showed a negative ellipticity at 222 nm, indicating a decreased triple helical content in these proteins and confirming the trypsin sensitivity.

Melting temperatures (T_m_) of purified collagens were determined by incremental heating with simultaneous measurement of the molar ellipticity at 222 nm. Melting curves of all purified collagen II proteins are depicted in Figure 2B using a curve fit model assuming that 100% of the molecules were folded initially. The curves for WT, R75C, R134C and R704C collagens show similar profiles, while R740C and R789C proteins showed a decreased T_m_ value indicating a decreased thermal instability as compared to other collagens. The calculated melting temperatures are summarized in Table 1.

### 2.3. Inhibition of MMP Activity Prevents Cleavage of R789C

**Figure 3:**
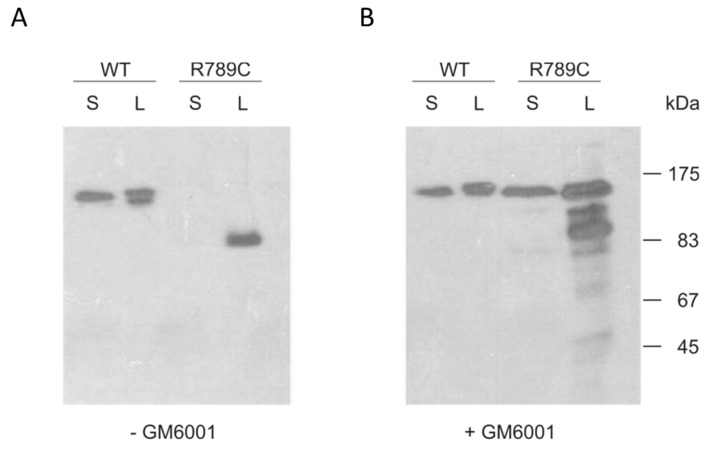
Analysis of proteolytic processing using the MMP inhibitor GM6001. Cells expressing wildtype (WT) and R789C collagens were cultured for two days in the absence (**A**) or presence (**B**) of the MMP inhibitor GM6001 (25 µM). Supernatants (S) and cell lysates (L) were harvested and analyzed by immunoblot with an antibody direct against the myc epitope. After treatment with GM6001, the R789C collagen was partially protected from degradation, indicating that the reduced mass and shift in mobility are caused by a proteolytic cleavage by MMPs.

To investigate if the shift in R789C protein mobility is due to MMP cleavage, cells expressing wild type and R789C collagen were incubated for two days with the MMP inhibitor GM6001. In the presence of GM6001, significant amounts of R789C proteins remained uncleaved, in contrast to what was seen in the absence of the inhibitor (Figure 3). This suggests that the mutation R789C increases the susceptibility and/or accessibility for MMPs to cleave collagen II. Interestingly, the resulting uncleaved R789C protein was now detected in the supernatant in large amounts indicating that inhibition of cleavage results in increased secretion. Even though some uncleaved protein was secreted, a large proportion of both uncleaved and cleaved collagen was still retained intracellularly.

### 2.4. Effect of Mutations on Intracellular Collagen Trafficking in Transfected HT1080 Cells

**Figure 4:**
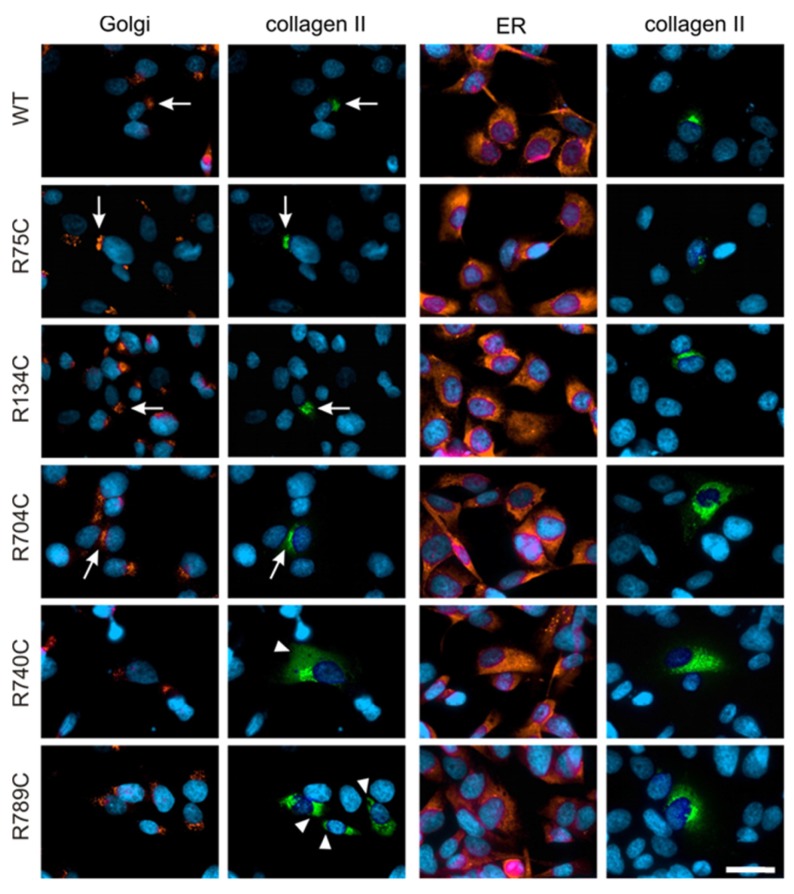
Immunofluorescence microscopy of transfected HT1080 cells showing the intracellular localization of wildtype and mutant collagen II variants. HT1080 cells transfected with collagen II constructs were analyzed three days post-transfection with antibodies directed against the myc epitope of collagen II (green) and either against PDI as a marker for ER or 58K as a marker for the Golgi apparatus (both in red). Colocalization of collagen with 58K in the Golgi compartment is seen in cells expressing WT, R75C, R134C and R704C collagen (arrows). In cells expressing R740C and R789C collagens, significant amounts of protein colocalize with PDI in the ER detected outside the Golgi apparatus (arrowheads). Scale bar, 25 µm.

Collagen II trafficking was visualized by co-staining with antibodies directed against PDI and the 58K protein as marker for the ER and the Golgi apparatus, respectively, in transfected HT1080 cells. WT, R75C, R134C and R704C collagen II proteins co-localized mainly with the perinuclear Golgi apparatus, demonstrating that the intracellular trafficking of these proteins from ER to Golgi apparatus is not affected by the mutation (Figure 4). In contrast, the mutant R740C and R789C were mainly detected in the ER, which is typically spread throughout the whole cell. Overlapping staining for collagen II and PDI in cells expressing R740C and R789C proteins suggests that the protein is retained in the ER compartment due to the mutation (Figure 4).

### 2.5. C-Terminal Mutations Induce Activation of the Unfolded Protein Response Due to Accumulation of Misfolded Collagen II Proteins

**Figure 5:**
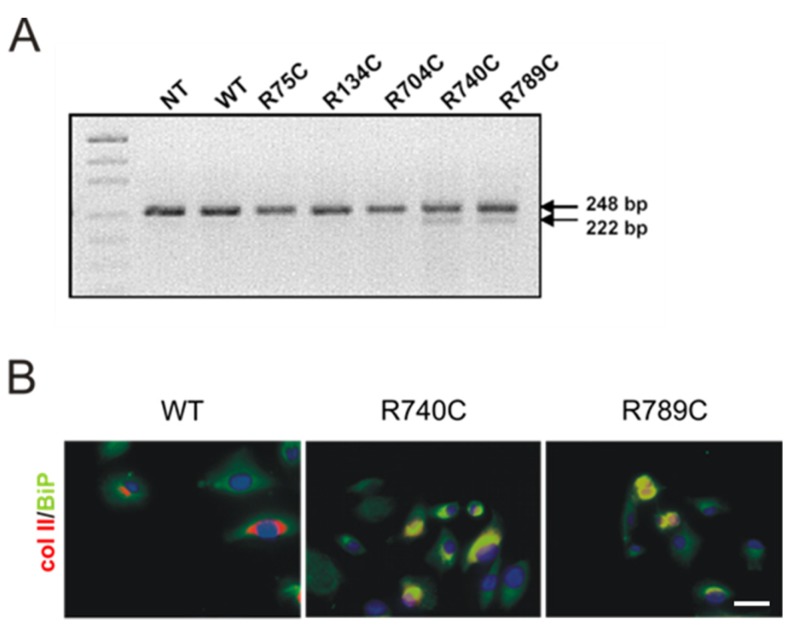
Detection of stress-induced XBP-1 splicing and BiP expression in transfected HT1080 cells. (**A**) mRNA isolated from HT1080 cells was used for RT-PCR with primers specific for XBP-1. The PCR products were separated on 2.5% agarose gels. XBP-1 mRNA (248 bp) could be detected in all cells. Splicing of XBP-1 due to ER stress gives rise to a 222-bp fragment that was seen only in cells transfected with R740C and R789C constructs. (**B**) HT1080 cells transfected with collagen II constructs were analyzed three days post-transfection by staining with antibodies directed against collagen (red) and BiP (green). BiP expression was observed in all the cells. However, increased BiP expression and colocalization with collagen II resulting in strong yellow signals were seen only in cells expressing R740C and R789C collagens. Scale bar, 25 µm.

Intracellular retention of misfolded proteins in the ER leads to activation of a complex signal transduction pathway called the unfolded protein response (UPR) [22,23]. UPR results in the induction of ER stress response genes by two potent transcription factors, activating transcription factor-6 and XBP-1 (X-box DNA binding protein-1). Active XBP-1 is generated by excision of a 26-nucleotide sequence from the XBP-1 transcript by IRE1 endonuclease in response to accumulation of misfolded proteins in the ER [22,23]. XBP-1 splicing was investigated by RT-PCR from RNA isolated from HT1080 cells using specific primers giving rise to a 248-bp band in the case of unspliced XBP-1 and to a 222-bp band where XBP-1 is spliced. Figure 5A shows the PCR products resolved on a 2.5% agarose gel. In cells expressing WT, R75C, R134C and R704C collagen, only the unspliced form of XBP-1 was detected, while in cells expressing R740C and R789C collagen, the spliced variant (XBP-S) could also be detected. This suggests that the intracellular retention of misfolded proteins has triggered an ER stress response.

#### Expression and Association of Chaperones with Mutant Collagen II Proteins

Activation of XBP-1 results in upregulation of ER-resident molecular chaperones. To investigate whether the splicing of XBP-1 associated with accumulation of R740C and R789C mutant collagen II proteins results in increased expression and association of BiP in transfected HT1080 cells, cells were stained for collagen II and BiP. BiP expression was observed in cells expressing WT collagen, and collagen II staining was observed around the Golgi compartment in the transfected cells (Figure 5B) with only little co-staining detected between BiP and collagen II. In cells expressing R740C and R789C collagens, significant co-staining of BiP and collagen II proteins was observed, and the intensity of BiP staining was also increased in these cells, compared to the cells transfected with wildtype collagen II.

### 2.6. Intracellular Accumulation of Misfolded Proteins Induces Apoptosis in Cells Expressing Mutant Collagens

Retention of large amounts of misfolded proteins affects cell viability. We therefore analyzed if HT1080 cells expressing mutant collagens undergo apoptosis as a consequence of ER stress. The executioner caspase caspase-3 has to be activated by other proteases like caspase-8 and -9. Caspase-3 then cleaves a large number of cellular proteins, and their degradation finally disrupts cellular homeostasis and causes cell death.

**Figure 6:**
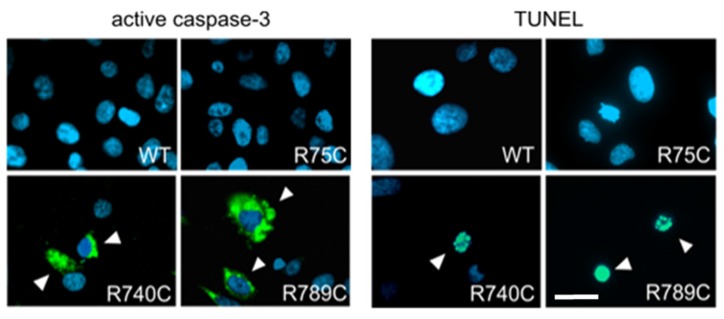
Detection of apoptosis in HT1080 cells transfected with collagen II constructs. HT1080 cells transfected with collagen II constructs were analyzed three days post-transfection for the presence of active caspase-3 (green) as a marker for apoptosis. Active caspase-3 was only found in cells expressing R740C and R789C collagens (left panel, indicated by arrow heads). Four days after transfection, HT1080 cells transfected with R740C and R789C constructs were positive for TUNEL staining (green, right panel, indicated by arrow heads). Nick labeling was neither seen in non-transfected cells, nor in cells transfected with other collagen constructs (Supplemental Figure S1). Scale bar, 25 µm.

Three days after transfection, R740C- and R789C-expressing HT1080 cells were positive for activated caspase-3 detected by immunostaining using a FITC-conjugated anti-active caspase-3 antibody. In non-transfected cells or cells expressing WT, R75C, R134C and R704C collagens, no active caspase-3 staining could be detected (Figure 6 and Supplemental Figure S1).

Thus, cells expressing R740C and R789C collagens become apoptotic, most likely due to an ER stress response triggered by irreversible accumulation of misfolded proteins. Apoptosis is accompanied by cleavage and fragmentation of nuclear DNA. We therefore analyzed if DNA fragmentation could be detected in HT1080 cells expressing R740C and R789C collagens. Nick-labeled DNA was not observed in non-transfected cells and cells transfected with wild type and R704C constructs, whereas nick-labeled DNA was observed in cells expressing R740C and R789C (Figure 6).

**Figure 7:**
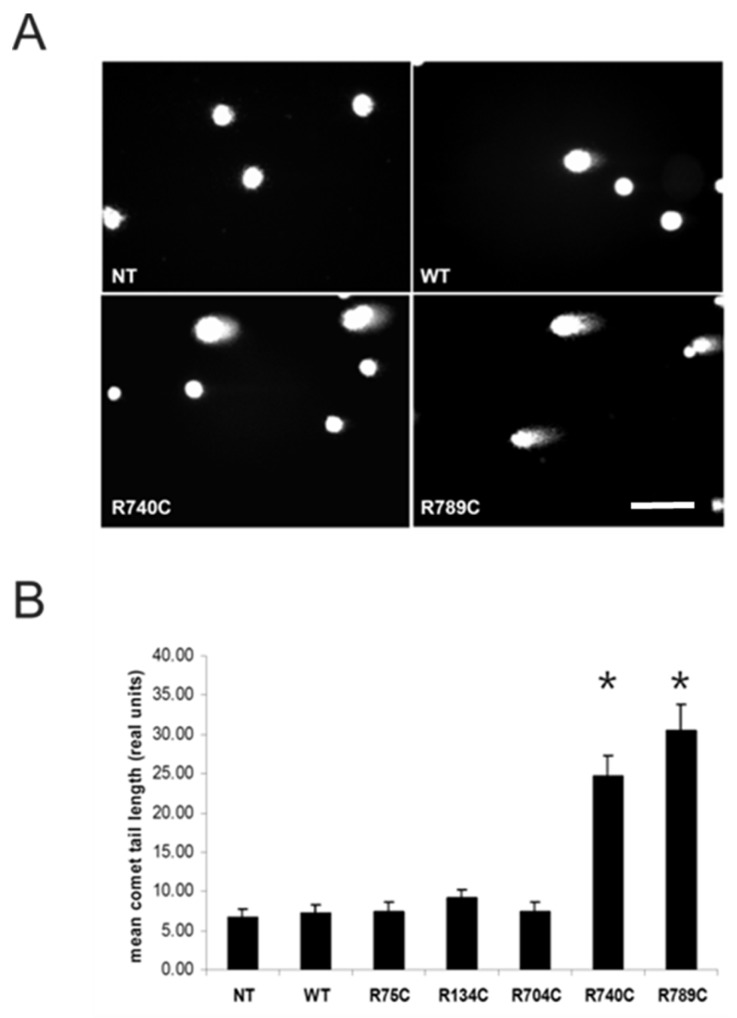
Single cell gel electrophoresis (comet assay) of HT1080 cells transfected with collagen II variants. (**A**) Nuclei of non-transfected (NT) and WT collagen-expressing cells were round when visualized after electrophoresis. In contrast, a comet-shaped appearance of fragmented nuclei was seen in cells transfected with R740C and R789C, indicating ongoing DNA fragmentation. Scale bar, 25 µm. (**B**) The mean tail length was evaluated for these comets (20 cells for each genotype) using the CometScore software. A significant increase in tail length was observed in cells transfected with R740C and R789C collagen constructs, while cells transfected with all other constructs behaved similar to non-transfected controls. Asterisks indicate significant difference compared to and WT collagen-expressing cells with *p* < 0.05.

Apoptosis was quantified in HT1080 cells four days post-transfection by comet assays (single cell gel electrophoresis) (Figure 7). Nuclear DNA fragmentation leads to DNA tailing that can be visualized by a comet-shaped appearance. Such comets tails were observed in cells transfected with R740C and R789C constructs (Figure 7A). From each genotype (20 cells per genotype), the mean tail length was determined using the CometScore software (Figure 7B). Apoptosis in cells expressing R740C and R789C collagens was indicated by a significant increase in tail length corresponding to DNA fragmentation.

## 3. Discussion

Point mutations in fibrillar collagens cause a number of abnormalities in connective tissues, leading for example to brittle bone disease, osteoarthritis and osteochondrodysplasias [24,25]. In the present study, a set of *COL2A1* mutations leading to substitution of an arginine to cysteine residue in the triple helix were studied. The mutations were selected based on their localization within the triple helix and position with the Gly-X-Y repeats. Interestingly, the selected mutations cause a rather heterogeneous disease spectrum in humans including Czech dysplasia metatarsal type (R75C), Stickler syndrome (R704C) and spondyloepiphyseal dysplasia congenital (R789C). We included two artificial mutations (R134C and R740C) and analyzed their effects on intracellular protein trafficking, secretion and cell survival.

We were able to express all variants in both 293 EBNA and HT1080 cells. WT, R75C, R134C and R704C proteins were mainly detected in the cell culture supernatant indicating a normal secretion. The variant R740C showed a retarded secretion with similar protein amounts in the supernatant and cell lysate, respectively. R789C collagen was not only retained intracellularly, but also processed, resulting in a prominent band at around 90–100 kDa. This cleavage took place already in the intracellular compartment. Altered secretion and moderate intracellular retention of R789C collagen was reported earlier when this construct was expressed in SW-1353 and HT1080 cells [26,27]. However, the processing was observed for the first time and is in contrast to earlier reports [27,28]. This apparent difference might be explained by expression levels depending on the vectors used and it is likely that a cell starts to degrade the protein when a certain amount has been accumulated. Even though two variants were not properly secreted, we were able to purify sufficient amounts of all constructs to perform biochemical analysis. CD spectroscopy with collagen-specific spectra indicated correct folding of the variants WT, R75C, R134C and R704C. The melting temperature of wild type collagen II with 38.6 °C was 2.4 °C lower than for collagen extracted from bovine nasal cartilage [29]. This difference in the absolute melting temperature might be caused by inefficient hydroxylation by 293 EBNA cells [30,31]. The R75C, R134C and R704C proteins had a 2.5 °C lower melting temperature than the wild type protein, suggesting slight structural differences even though these changes were not pronounced enough to loose trypsin resistance. In contrast and in agreement with earlier studies [26,27], R740C and R789C collagens had a significantly reduced melting temperature, and incubation with trypsin led to complete degradation, confirming an unstable triple helix in these variants. Such an instability could well contribute to the disease mechanism in human patients and has been reported to be involved for mutations in *COL2A1* causing Kniest dysplasia [32], in *COL3A1* causing Ehlers-Danlos syndrome type IV [33] and in COL17A1 causing junctional epidermolysis bullosa [34].

The cleavage of partially-misfolded R789C collagen might be due to an increased accessibility for proteases at regions around the site of mutation. MMP-1, MMP-8 and MMP-13 [35,36] are able to initiate the intrahelical cleavage of triple helical collagen at neutral pH. These collagenases cleave the collagens types I, II and III specifically at a single site (Gly_775_–Leu/Ile_776_) within each α chain of the triple helical collagen molecule [37]. The exact cleavage position in the R789C protein is yet to be identified, but it is attractive to speculate that the mutation in close proximity of the well-known MMP cleavage site facilitates cleavage due to local unfolding [38,39,40]. The fact that pretreatment of the cells with the MMP inhibitor GM6001 abrogated the processing further supports the notion that MMPs are responsible for the cleavage. Interestingly, the inhibition of cleavage leads to an increased secretion of the now fully-intact protein into the supernatant. This is in good agreement with earlier studies in which mutating the MMP cleavage site resulted in an increased secretion of R789C collagen [27,28,41]. Increased collagen cleavage by MMPs at this position has indeed been shown in patients with specific forms of osteoarthritis, as well as in transgenic mice with osteoarthritis [42,43,44]. This might explain why specific collagen mutations predispose for cartilage degeneration. 

The fibroblastic cell line HT1080 is commonly used to study protein expression, trafficking and secretion of collagen II [45,46]. Immunoblotting already suggested that a considerable amount of R740C and R789C protein was present in cell lysates owing to intracellular retention. Co-staining with compartment-specific antibodies revealed that WT, R75C, R134C and R704C proteins were found mainly in the Golgi compartment, indicating that these proteins are being transported efficiently to the extracellular space. In contrast, R740C and R789C proteins showed only weak co-localization with the 58k Golgi marker and were instead detected in other cellular compartments. Co-staining with PDI, an ER-resident chaperone, suggests that mutant and misfolded R740C and R789C proteins are retained in the ER [47,48]. A similar intracellular retention of the misfolded proteins in the ER due to point mutations was reported for other cartilage proteins such as COMP, collagen IX and matrilin-3 in patients and animal models [49,50].

The retention of misfolded protein in the ER leads to the activation of a complex signal transduction pathway called the unfolded protein response (UPR) [22,23]. The fact that a targeted induction of ER stress alone is able to induce cartilage pathology [51] underlines the crucial role of this process in disease initiation. The splicing of XBP-1 mRNA is a hallmark of the mammalian UPR, and we indeed detected this unconventional splicing event in cells expressing R740C and R789C collagen. A similar observation was made in response to misfolded protein accumulation due to a mutation in the NC1 domain of collagen X [52]. XBP-1 splicing in turn leads to an upregulation of ER-resident molecular chaperones like BiP [53]. We also detected an upregulation and colocalization of BiP with R740C and R789C procollagen chains, and similar findings were reported for collagen chains harboring mutations in type I collagen from patients with osteogenesis imperfecta [54].

Prolonged accumulation of misfolded proteins in the ER without degradation can lead to programmed cell death [55,56]. Indeed, the large amount of rounded and dead cells observed in cultures expressing R740C and R789C collagens may indicate that the cells are undergoing apoptosis. We were able to detect an increase of active caspase-3 and TUNEL staining in cells transfected with R740C and R789C constructs, confirming earlier observations [57]. An increased apoptosis may also explain the reduced number of chondrocytes observed in both patients and animal models [37,58,59]. In addition to intracellular disturbances described above, changes in the ECM might also contribute to the phenotype. It has been shown earlier for mutations in COMP that both intra- and extra-cellular pathways are involved in the pathogenesis of pseudoachondroplasia [60] and that disruption of the ECM structure may cause phenotypes even in the absence of impaired secretion [61]. The structure of the ECM could be affected by reduced amounts, the complete absence or the presence of mutated collagen II. The amount and quality of extracellular collagen II depends on the specific mutation, and this might explain why different mutations cause such a spectrum of disease phenotypes. 

In our study, mutations at the N-terminus (R75C, R134C) of the triple helix resulted in less pronounced effects on all parameters investigated compared to mutations at the C-terminus. Similar findings were reported for mutations in collagen I in which glycine substitutions towards the C-terminus of the collagen I chains are clinically more severe than those towards the N-terminus [62]. This might be due to the fact that triple helix formation and propagation initiates at the C-terminus, and mutations at this structurally-important site will interfere with protein stability [63,64,65]. Our findings suggest that the phenotype of patients harboring the C-terminal mutation R789C in the collagen II chain might be caused by accumulation of the mutated collagen II protein in the ER, leading to ER-stress and apoptosis of chondrocytes. However, this seems not to be a universal disease mechanism common for all collagen mutations. According to our study, even the rather C-terminal R704C and also the N-terminal R75C mutation neither induce ER stress nor apoptosis and might cause a disease predominantly via extracellular interference. In addition, it is tempting to speculate that other factors such as genetic modifiers or the presence of additional mutations in other gene loci yet to be identified contribute to a chondrodysplasia phenotype. Similar observations were made in patients with multiple epiphyseal dysplasia (MED) [66].

## 4. Materials and Methods 

### 4.1. Collagen II cDNA, Site-Directed Mutagenesis and Removal of Endogenous Signal Peptide

Human collagen II cDNA of 4.5 kb including the poly-adenylation signal was kindly provided by Fibrogen Europe. The eukaryotic expression vector pCEP-Pu containing a BM-40 signal peptide, puromycin resistance [67] and an N-terminal sequence coding for the his_6_-myc tag [68] was used for expression of collagen II variants in the mammalian cells. Site-directed mutagenesis was carried out using the XL Quick Change mutagenesis kit (Stratagene, La Jolla, CA, USA). Mutations were introduced into collagen II cDNA resulting in exchange of R75C, R134C, R704C, R740C and R789C. The primer pairs that were used for sited-directed mutagenesis are represented in Table 2. Nucleotide change leading to mutation is indicated in bold. The mutated constructs were inserted into the episomal expression vector pCEP-Pu- his_6_-myc tag (N-terminal) in-frame with the sequence of the signal peptide of BM-40 as described earlier [66].

### 4.2. Cell Culture and Transfection

Human embryonic kidney-derived 293 EBNA cells (Invitrogen) and human fibrosarcoma-derived HT1080 (CCL-121 from ATTC) cells were cultured in DMEM-F12 medium containing 200 U/mL penicillin, 200 µg/mL streptomycin, 20 mM l-glutamine and 10% FBS (Biochrom, Berlin, Germany), from now on referred to as standard medium, at 37 °C in a humified incubator with a 5% CO_2_ atmosphere. One hundred micrograms per milliliter of ascorbate were added to the cell culture medium during expression of recombinant collagens. Cells were transfected using FuGene6 reagent (Roche, Munich, Germany) following manufacturers instruction.

### 4.3. Purification of Collagen II Proteins

After 24 h post-transfection of 293 EBNA cells with collagen II constructs, cells were selected using puromycin, and the supernatants were harvested from stably-transfected cells and the secreted proteins purified on a nickel column by ion exchange chromatography, as previously described in [68].

### 4.4. CD Spectroscopy and Melting Curves 

CD spectroscopy was performed with purified collagen II proteins, which were dialyzed in 100 mM acetic acid, and the concentration was adjusted to 60 µg/mL. Two hundred microliters of collagen solution were used, and a spectrum between 190 nm and 280 nm was recorded using a Jasco J-715 Polarimeter at 4 °C. Melting curves were determined at 222 nm in the temperature range of 10–55 °C at increments of 1 °C/min.

### 4.5. SDS-PAGE and Immunoblotting

SDS polyacrylamide gel electrophoresis was performed using the buffer system of Laemmli. Protein samples were mixed with an equal volume of 2× SDS sample buffer (50 mM Tris-HCl, pH 6.8, 2% (*w*/*v*) SDS, 20% glycerol and 0.025% (*w*/*v*) bromophenol blue), whereas cells were lysed and resuspended in 1× SDS sample buffer. Before loading, samples were reduced and denatured by adding 5% β-mercaptoethanol and boiled at 95 °C for 5 min. Equal amounts of supernatants and cell lysates obtained 72 h after transfection were resolved on 8% (*w*/*v*) SDS-PAGE gels and were analyzed by immunoblotting. After SDS-PAGE, proteins were transferred to nitrocellulose and incubated for 1 h in TBS in the presence of 5% (*w*/*v*) milk protein. Rabbit anti-myc antibodies or goat anti-collagen II antibodies were used at a dilution of 1:1500, and the bound primary antibodies were detected using a 1:2000 dilution of horseradish peroxidase-conjugated rabbit anti-goat or swine anti-rabbit IgG (Dako Corp., Glostrup, Denmark). Blots were developed with a self-made enhanced chemiluminescence (ECL) reaction.

### 4.6. Trypsin Digestion

Protease treatment of purified collagen II variant proteins (3 micrograms) was carried out in a 50-µL reaction volume containing digestion buffer (50 mM Tris-HCl, pH 7.4, containing 150 mM NaCl and 10 mM EDTA). Trypsin was added to the samples at a concentration of 100 µg/mL and was incubated for 2 min at 25 °C. The digestion was stopped by addition of soybean trypsin inhibitor (Sigma, Munich, Germany) at a final concentration of 5 µg/mL. SDS-PAGE sample buffer containing 5% β-mercaptoethanol was added, and the samples were resolved on 8% SDS-PAGE. The resolved proteins were visualized by silver staining.

### 4.7. Inhibition of Matrix Metalloproteinase Cleavage 

HT1080 cells were transfected with collagen II constructs, and three days after transfection, the supernatants were collected. Fresh culture medium was supplemented with 25 µM GM6001 (1 mM stock in DMSO) and 100 µg/mL of ascorbate. Supernatants and cell lysates were harvested 48 h after addition of the inhibitor. Proteins were resolved by SDS-PAGE on 8% gels and collagen II, and after blotting, collagen II and fragments thereof were detected using the anti-myc antibody.

### 4.8. Reverse Transcription-PCR for Analysis of Spliced XBP1 Transcripts 

RNA was extracted using the TRIZOL reagent (Invitrogen, Carlsbad, CA, USA) according to the manufacturer’s instruction from cells 72 h post-transfection. To avoid ER stress induced by glucose starvation, fresh medium with serum was added 2 h prior to RNA extraction. One hundred nanograms of total RNA/reaction were used for cDNA synthesis using reverse transcriptase and random hexamers (Roche Diagnostics, Basel, Switzerland). XBP1 transcripts were analyzed by PCR of the cDNA using primers corresponding to the spliced and unspliced forms of the XBP1 transcripts (5′-GGAGTTAAGACAGCGCTTGG-3′, bp 401–420) and antisense (5′-ACTGGGTCCAAGTTGTCCAG-3′, bp 648–629) primers spanning the XBP1 RNA processing sequence (GenBank Accession Number AB076383) [37], The PCR products corresponding to unspliced and spliced *XBP1* (248 and 222 bp, respectively) were obtained after 35 cycles using a primer annealing temperature of 60 °C. The products were resolved on 2.5% (*w*/*v*) agarose gels and visualized under ultraviolet light.

### 4.9. Immunofluorescence Staining of HT1080 Cells

Transfected and non-transfected cells were grown on glass cover slips in 24-well dishes. After 3 days of transfection, the cells were fixed with 2% paraformaldehyde and permeabilized using 0.2% Triton X-100 in PBS. Cells were blocked with 10% normal goat serum in PBS after three washes with PBS. Primary antibodies were added at a dilution of 1:1000 and incubated for 60 min, followed by washings to remove unbound antibodies. Bound primary antibodies were detected with a secondary fluorescent-labelled antibody for a further 60 min. Antibodies directed against protein disulfide isomerase (PDI) (Biomol, Hamburg, Germany) and the 58K protein Sigma (Munich, Germany) were used as markers for the ER and the Golgi apparatus, respectively. Mouse anti-myc antibodies were used to detect the myc epitope of recombinantly-expressed collagen II. Rabbit anti-BiP was used for the detection of BiP. Secondary anti-rabbit and secondary anti-mouse Cy3- and Alexa488-conjugated antibodies were from Molecular Probes (Leiden, The Netherlands). Nuclei were stained with bisbenzimide (Sigma, 0.1 µg/mL). The slides were finally mounted in DAKO fluorescent mounting medium and examined under an Axiophot fluorescence microscope (Zeiss, Oberkochen, Germany).

### 4.10. Nick Labelling 

TUNEL staining was performed to detect apoptosis using the DeadEnd™ Fluorometric TUNEL System (Promega, Madison, WI, USA) following a slightly modified manufacturer’s protocol. Cells were fixed by 25-min immersion in 4% paraformaldehyde solution four days post-transfection. After two washes in PBS for 5 min cells were permeabilized with 0.2% Triton X-100 solution in PBS for 5 min. After rinsing cells in PBS, 100 μL of equilibration buffer were added for 5–10 min at room temperature. Cells were then incubated with 50 μL of reaction buffer (rTdT incubation buffer) for 60 min inside a dark humidified chamber at 37 °C. The tailing reaction was terminated by adding 2× saline sodium citrate solution (SSC) for 15 min. Nuclei were stained with bisbenzimide (0.1 µg/mL in PBS) for 5 min. After washing with deionized water for 5 min at room temperature, cover slips were mounted on histo-slides with DAKO mounting medium.

### 4.11. Comet Assay or Single Cell Gel Electrophoresis 

Four days after transfection, cells were trypsinized, counted and diluted to give approximately 5 × 10^4^ cells/mL. Eighty microliters of the cell suspension were added to 400 µL of 0.5% low melting agarose maintained at 37 °C, and 90 µL of this suspension were pipetted onto a slide, which was precoated with 1.0% agarose. An additional 1% low melting agarose layer without cells was added after solidification of the layer. Alkaline lysis solution (2.5 M NaCl, 100 mM EDTA, 10 mM Tris-HCl, 1% Triton X-100, 10% DMSO, pH to 10.0) was added to the slides at 4 °C for 1 h in the dark to lyse cells. After lysis, the slides were placed in neutralizing solution (400 mM Tris-HCl, pH 7.5) and rinsed three times for 30 min to remove salts and detergents. Slides were then placed in a horizontal electrophoresis chamber with alkaline buffer (300 mM NaOH, 1 mM EDTA, pH > 13) and incubated for 20–60 min to allow unwinding of the DNA. Electrophoresis of the slides was run for 30 min at 0.6 V/cm. After electrophoresis, the slides were rinsed with neutralization buffer twice and stained with 100 µL ethidium bromide solution (10 mg/mL). Slides were scored immediately or alternatively dried in cold 100% ethanol before storage. DNA tailing due to fragmentation was visualized as comets using an inverted fluorescence microscope and evaluated using TriTek CometScore™ software (TriTek Corp, Sumerduck, VA, USA).

## Figures and Tables

**Table 1:** Melting temperatures (Tm) of the purified collagen II variants.

Protein	Melting Temperature (T_m_)
WT	38.6 °C
R75C	36.2 °C
R134C	36.1 °C
R704C	36.1 °C
R740C	30.2 °C
R789C	31.5 °C

**Table 2:** Primer pairs used for site directed mutagenesis.

Primer	Sequence
75F	5′-GGTCCTCAGGGTGCT**T**GTGGTTTCCCAGG-3′
75R	5′-CCTGGGAAACCAC**A**AGCACCCTGAGGACC-3′
134F	5′-CTGGTGAAAGAGGA**T**G**C**ACTGGCCCTGCTG-3′
134R	5′-CCAGCAGGGCCAGT**G**C**A**TCCTCTTTCACC-3′
704F	5′-GGAGCTGCTGGG**T**GCGTTGGACCCCC-3′
704R	5′-GGGGGTCCAACGC**A**CCCAGCAGCTCC-3′
740F	5′-CCCCCTGGC**T**G**C**GCTGGTGAACCCGG-3′
740R	5′-GGGTTCACCAGC**G**C**A**GCCAGGGGGG-3′
789F	5′-GGTCTGCCTGGGCAA**T**GTGGTGAGAGAGGATTCC-3′
789R	5′-GGAATCCTCTCTCACCAC**A**TTGCCCAGGCAGACC-3′

Numbers 75, 134, 704, 740 and 789 stand for the amino acid residue numbered from the start of the triple helix where the mutation of interest is located. Nucleotides marked in bold indicate the nucleotide change to introduce the desired mutation.

## Supplementary Materials

Supplementary materials can be found at http://www.mdpi.com/1422-0067/19/2/541/s1.
