# Supplementary material for: Impact of Arginine to Cysteine Mutations in Collagen II on Protein Secretion and Cell Survival
Source: Int J Mol Sci. 2018 Feb 11;19(2):541. doi: 10.3390/ijms19020541 (PMC5855763; doi:10.3390/ijms19020541)
Supplement: Supplementary file 1 [file ijms-19-00541-s001.pdf]

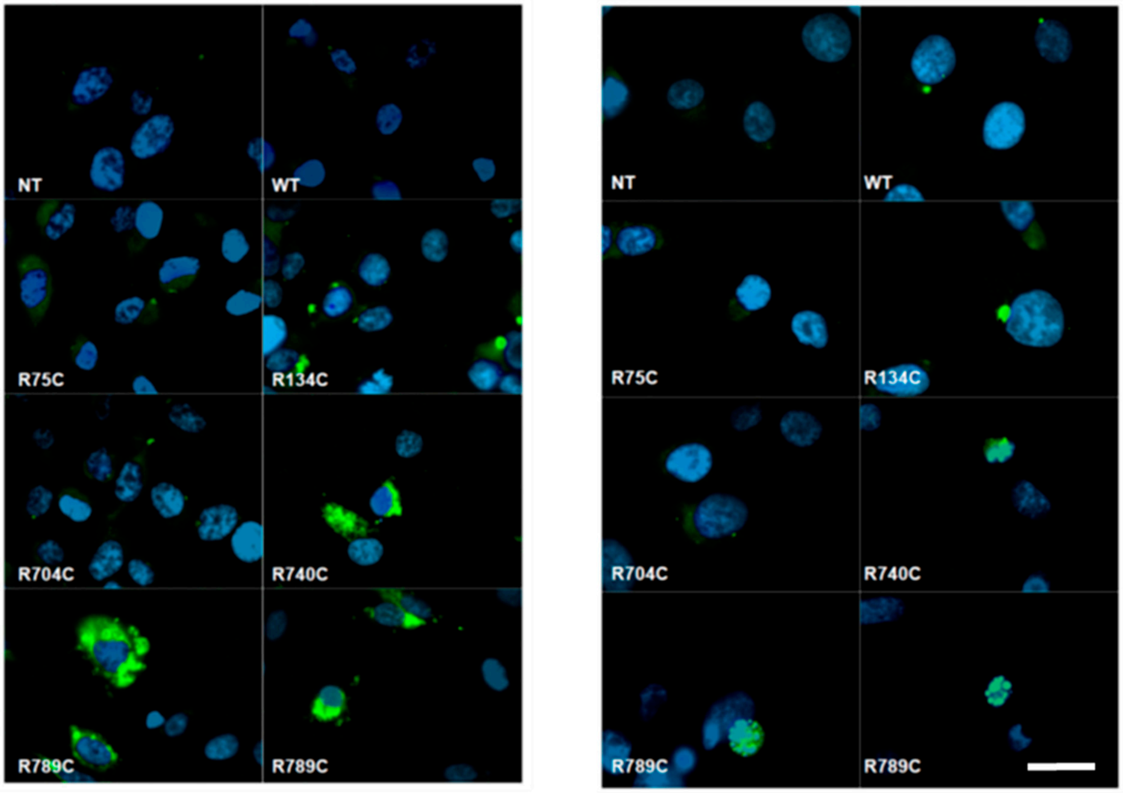

Figure S1. Detection of apoptosis in HT1080 cells transfected with collagen II constructs. HT1080 cells transfected with collagen II constructs were analyzed three days post transfection for the presence of active caspase-3 (green) as a marker for apoptosis. Active caspase-3 was only found in cells expressing R740C and R789C collagens (left panel, indicated by arrow heads). Four days after transfection, HT1080 cells transfected with R740C and R789C constructs were positive for TUNEL staining (green, right panel, indicated by arrow heads). Nick labeling was neither seen in non-transfected cells (NT) nor in cells transfected with other collagen constructs. Scale bar, 25 µm.
